# Reconstruction and computer analysis of the structural and functional organization of the gene network regulating cholesterol biosynthesis in humans and the evolutionary characteristics of the genes involved in the network

**DOI:** 10.18699/vjgb-24-94

**Published:** 2024-12

**Authors:** A.D. Mikhailova, S.A. Lashin, V.A. Ivanisenko, P.S. Demenkov, E.V. Ignatieva

**Affiliations:** Novosibirsk State University, Novosibirsk, Russia; Novosibirsk State University, Novosibirsk, Russia Institute of Cytology and Genetics of the Siberian Branch of the Russian Academy of Sciences, Novosibirsk, Russia Kurchatov Genomic Center of ICG SB RAS, Novosibirsk, Russia; Novosibirsk State University, Novosibirsk, Russia Institute of Cytology and Genetics of the Siberian Branch of the Russian Academy of Sciences, Novosibirsk, Russia Kurchatov Genomic Center of ICG SB RAS, Novosibirsk, Russia; Novosibirsk State University, Novosibirsk, Russia Institute of Cytology and Genetics of the Siberian Branch of the Russian Academy of Sciences, Novosibirsk, Russia Kurchatov Genomic Center of ICG SB RAS, Novosibirsk, Russia; Novosibirsk State University, Novosibirsk, Russia Institute of Cytology and Genetics of the Siberian Branch of the Russian Academy of Sciences, Novosibirsk, Russia

**Keywords:** cholesterol biosynthesis, transcription factors, SREBP, gene networks, feedback loops, evolution, phylostratigraphy, gene age, биосинтез холестерина, транскрипционные факторы, SREBP, генные сети, регуляторые обратные связи, эволюция, филостратиграфия, возраст гена

## Abstract

Cholesterol is an essential structural component of cell membranes and a precursor of vitamin D, as well as steroid hormones. Humans and other animal species can absorb cholesterol from food. Cholesterol is also synthesized de novo in the cells of many tissues. We have previously reconstructed the gene network regulating intracellular cholesterol levels, which included regulatory circuits involving transcription factors from the SREBP (Sterol Regulatory Element-Binding Proteins) subfamily. The activity of SREBP transcription factors is regulated inversely depending on the intracellular cholesterol level. This mechanism is implemented with the participation of proteins SCAP, INSIG1, INSIG2, MBTPS1/S1P and MBTPS2/S2P. This group of proteins, together with the SREBP factors, is designated as “cholesterol sensor”. An elevated cholesterol level is a risk factor for the development of cardiovascular diseases and may also be observed in obesity, diabetes and other pathological conditions. Systematization of information about the molecular mechanisms controlling the activity of SREBP factors and cholesterol biosynthesis in the form of a gene network and building new knowledge about the gene network as a single object is extremely important for understanding the molecular mechanisms underlying the predisposition to diseases. With a computer tool, ANDSystem, we have built a gene network regulating cholesterol biosynthesis. The gene network included data on: (1) the complete set of enzymes involved in cholesterol biosynthesis; (2) proteins that function as part of the “cholesterol sensor”; (3) proteins that regulate the activity of the “cholesterol sensor”; (4) genes encoding proteins of these groups; (5) genes whose transcription is regulated by SREBP factors (SREBP target genes). The gene network was analyzed and feedback loops that control the activity of SREBP factors were identified. These feedback loops involved the PPARG, NR0B2/SHP1, LPIN1, and AR genes and the proteins they encode. Analysis of the phylostratigraphic age of the genes showed that the ancestral forms of most human genes encoding the enzymes of cholesterol biosynthesis and the proteins of the “cholesterol sensor” may have arisen at early evolutionary stages (Cellular organisms (the root of the phylostratigraphic tree) and the stages of Eukaryota and Metazoa divergence). However, the mechanism of gene transcription regulation in response to changes in cholesterol levels may only have formed at later evolutionary stages, since the phylostratigraphic age of the genes encoding the transcription factors SREBP1 and SREBP2 corresponds to the stage of Vertebrata divergence.

## Abstract (ru)

Холестерин – это незаменимая структурная компонента клеточных мембран, предшественник витамина D и стероидных гормонов. У человека и других видов животных холестерин поступает в организм с пищей, а также синтезируется в клетках многих тканей de novo. Ранее нами была реконструирована генная сеть регуляции внутриклеточного уровня холестерина, включавшая регуляторные контуры, функционирующие при участии транскрипционных факторов подсемейства SREBP (sterol regulatory element-binding proteins). Активность транскрипционных факторов подсемейства SREBP регулируется в обратной зависимости от уровня холестерина в клетке. Этот механизм реализуется при участии белков «холестеринового сенсора», включающего белки SCAP, INSIG1, INSIG2, MBTPS1/S1P, MBTPS2/S2P и транскрипционные факторы подсемейства SREBP. Повышенный уровень холестерина является фактором риска сердечно-сосудистых заболеваний, а также сопутствующим фактором многих патологических состояний. Систематизация сведений о молекулярных механизмах, контролирующих активность факторов подсемейства SREBP и биосинтез холестерина, в формате генной сети и получение новых знаний о генной сети как едином объекте чрезвычайно важны в контексте понимания молекулярных механизмов развития заболеваний. Средствами компьютерной системы ANDSystem нами построена генная сеть регуляции биосинтеза холестерина в клетке. Генная сеть включает данные: (1) о ферментах, осуществляющих биосинтез холестерина; (2) белках, функционирующих в составе «холестеринового сенсора»; (3) белках, регулирующих активность белков «холестеринового сенсора»; (4) генах, кодирующих белки этих групп; (5) генах, транскрипция которых регулируется при участии транскрипционных факторов подсемейства SREBP (генах-мишенях). Проведен анализ генной сети и выявлены замкнутые регуляторные контуры, контролирующие активность транскрипционных факторов подсемейства SREBP. Эти контуры реализуются с участием генов PPARG, NR0B2/ SHP1, LPIN1, AR и кодируемых ими белков. Исследование филостратиграфического возраста генов показало, что предковые формы большинства генов человека, кодирующих ферменты биосинтеза холестерина и белки «холестеринового сенсора», могли возникнуть на достаточно ранних эволюционных этапах (Cellular organisms (корень филостратиграфического дерева) и этапах дивергенции Eukaryota и Metazoa). Однако механизм регуляции транскрипции генов в ответ на изменение уровня холестерина мог сформироваться только на более поздних эволюционных этапах, поскольку филостратиграфический возраст генов транскрипционных факторов подсемейства SREBP соответствует более позднему этапу эволюции (стадии дивергенции Vertebrata).

## Introduction

Cholesterol is an important substance in the animal body. It is present in all tissues as part of cell membranes, stabilizing the membrane structure (Koolman, Roehm, 2005). With an increase in cholesterol content, the membrane becomes more densely packed, contains fewer cavities, due to which its permeability to small molecules, including oxygen, decreases. This mechanism contributed to the adaptation of organisms to an oxygen-rich atmosphere, and, as a result, the protection of cells from oxidative stress (Zuniga-Hertz, Patel, 2019). It is noteworthy that cholesterol is not synthesized in fungi and plants, and the cell membrane of these organisms contains compounds similar in structure – ergosterol (in fungi) and β-sitosterol and stigmasterol (in plants) (Desmond, Gribaldo, 2009; Ferrer et al., 2017; Choy et al., 2023).

In animals, cholesterol has other important functions. This substance is a precursor of bile acids and steroid hormones (progesterone, estradiol, testosterone, calcitriol, cortisol) (Luo et al., 2020; Schade et al., 2020).

In humans and other animal species, cholesterol enters the body with food, and is also synthesized in the cells of many tissues de novo (Luo et al., 2020). The initial metabolites for cholesterol synthesis are acetyl-CoA and acetoacetyl-CoA, and more than 20 enzymes are involved in the biosynthesis process (Desmond, Gribaldo, 2009; Nes, 2011). Intermediate metabolites of the cholesterol biosynthesis pathway, such as geranylgeranyl pyrophosphate and farnesyl pyrophosphate, can also play an important role in animal cells. These metabolites are substrates in prenylation reactions. Prenylation is a common covalent post-translational modification of various proteins. Proteins that undergo prenylation include, for example, Ras and small GTP-binding proteins (GTPases). Such post-translational prenylation is important for the proper localization and activation of proteins (Waller et al., 2019).

**Fig. 1.**
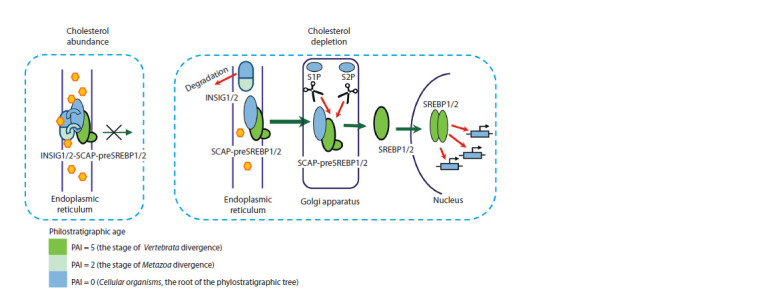
The functioning of the “cholesterol sensor”. Yellow hexagons represent cholesterol molecules; INSIG1/2 – endoplasmic reticulum anchor proteins INSIG1 and INSIG2; SREBP1/2 – transcription factors SREBP1 and SREBP2; preSREBP1/2 – preSREBP1 and preSREBP2, which are inactive precursor proteins of SREBP1 and SREBP2; SCAP – SREBF chaperone protein interacting with preSREBP1 and preSREBP2; S1P and S2P proteins are proteases that are encoded by the MBTPS1 and MBTPS2 genes (respectively). The colors of the objects correspond to the phylostratigraphic age of the genes, which was estimated based on the PAI (the procedure for calculating PAI is described in the “Materials and methods” section). At high cholesterol levels (the left part of the Figure), cholesterol stabilizes the structure of INSIG1 and INSIG2 (designated as INSIG1/2), increasing its affinity for SCAP. The anchor proteins INSIG1 and INSIG2 help the SCAP-preSREBP1/2 complex to be preserved on the ER membrane. In cholesterol-deprived cells (the right part of the Figure), the reduction of sterol leads to ubiquitination and rapid degradation of INSIG1/ 2. The binding of SCAP to INSIG1/2 is destabilized. This gives the SCAP-preSREBP1/2 complex an opportunity to escape ER. The SCAPpreSREBP1/ 2 complex is transported to the Golgi apparatus, where the preSREBP1/2 proteins are cleaved by the S1P and S2P proteases. As a result of cleavage of the preSREBP1 and preSREBP2 proteins, active transcription factors SREBP1 and SREBP2 (designated as SREBP1/2) are formed. The description of the scheme is based on publications (DeBose-Boyd, Ye, 2018; Jiang et al., 2020).

Earlier, a gene network regulating intracellular cholesterol level was built, and four feedback loops involving transcription factors from the sterol regulatory element-binding protein subfamily (SREBP1 and SREBP2) were identified (Kolchanov et al., 2013; Merkulova et al., 2013). In the cells of animal organisms, there is a mechanism regulating the activity of transcription factors from the SREBP subfamily depending on cholesterol level (DeBose-Boyd, Ye, 2018; Jiang et al., 2020). This mechanism involves a number of proteins, which, in combination with transcription factors from the SREBP subfamily, will be further referred to as the “cholesterol sensor”. A diagram showing how the “cholesterol sensor” functions is given in Figure 1.

The fuctioning of SREBPs can also be regulated in response to external signals affecting the cell, for example, insulin and growth factors (Sundqvist et al., 2005; Arito et al., 2008; Peterson et al., 2011). Due to regulation of this kind, fine-tuning of the SREBPs activity is carried out depending on the state of the cell and the organism as a whole. In turn, SREBPs control the expression of proteins involved in the regulation of a large number of cellular functions, integrating local gene networks that control various biological processes (Jeon, Osborne, 2012).

Elevated cholesterol levels are a risk factor for the development of cardiovascular diseases (atherosclerosis, coronary heart disease) (VargasAlarcon et al., 2019; Macvanin et al., 2024), and can also act as a concomitant factor in obesity (Kim et al., 2010), diabetes (Zhang F. et al., 2018), non-alcoholic fatty liver disease, non-alcoholic steatohepatitis (Li et al., 2023), hepatocarcinoma (Paul et al., 2022), tumor processes (Jiang et al., 2020) and inflammation (Shimano, Sato, 2017). Obtaining new knowledge about the gene network regulating cholesterol biosynthesis, as a single object, is extremely important in the context of understanding the connection of this system with diseases.

The aim of this study is to systematize data on the molecular mechanisms controlling the activity of transcription factors of SREBP subfamily and mechanisms controlling cholesterol biosynthesis using the format of a gene network and subsequent analysis of the structural and functional organization of the network and analysis of the evolutionary characteristics of the genes involved in it.

## Materials and methods

**Lists of genes used for building the gene network. **The list comprising 24 human genes encoding enzymes of cholesterol biosynthesis (Supplementary Material 1)1 was compiled based on data from WikiPathways (Agrawal et al., 2024).


Supplementary Materials are available in the online version of the paper: https://vavilov.elpub.ru/jour/manager/files/Suppl_Mikhailova_Engl_28_8.pdf


The list, which included seven genes encoding proteins of the “cholesterol sensor” (Supplementary Material 2) was formed based on the description of the mechanism regulating activity of SREBP1 and SREBP2 according to data given in publications (DeBoseBoyd, Ye, 2018; Jiang et al., 2020).

The list containing 31 human genes, the transcription of which is regulated by factors of the SREBP subfamily (SREBP1 or SREBP2 target genes), was formed based on data from TRRD (Kolchanov et al., 2002) and TRRUST (https://www.grnpedia.org/trrust/) (Han et al., 2018). The final version of the list of SREBP target genes (Supplementary Material 3) included genes for which data on associations with SREBP1 or SREBP2 were found in ANDSystem (Ivanisenko et al., 2019).

The list of genes encoding proteins regulating the activity of proteins and genes of the “cholesterol sensor” (“regulatory proteins”) (Supplementary Material 4) was formed using ANDSystem (Ivanisenko et al., 2019). “Regulatory proteins” were found using ANDVisio (ANDSystem software component) with the help of the built-in Pathway wizard tool. The associations between the “regulatory proteins” and proteins or genes of the “cholesterol sensor” obtained in this way were verified manually.

**Building the gene network regulating cholesterol biosynthesis.** The construction of the gene network was carried out using ANDSystem (Ivanisenko et al., 2019). In the first step, we built gene networks that included small groups of genes (hereinafter referred to as “small gene networks”). The procedures for building “small networks” are described in Supplementary Material 5. The number of objects in the networks is given in Supplementary Material 6. These “small networks” were then merged together in the ANDVisio tool applying the “Union of graphs” command. We merged “small networks” that included the following associations: (1) between the “regulatory proteins” and genes and proteins of the “cholesterol sensor”; (2) between SREBPs and target genes, and between target genes and the encoded proteins; (3) between proteins encoded by SREBP target genes, and genes and proteins of the “cholesterol sensor”; (4) between genes or proteins of the “cholesterol sensor” (with the exception of SREBPs) and the SREBF1, SREBF2 genes and the encoded proteins; (5) between enzymes of cholesterol biosynthesis and cholesterol.

**Search for feedback loops. **The feedback loops that included 3, 4 or 5 objects, among which were factors SREBP1 and SREBP2, were found with the help of the ANDVisio built-in Pathway wizard tool. The search was performed based on the templates presented in Supplementary Material 7. According to the length of the template (which was equal to the number of objects involved in feedback loops), the number and types of intermediate objects were specified. The pathways found in this way were expanded by adding interactions between genes and the encoded proteins (“expression” type interactions), thus obtaining closed regulatory circuits.

**Identification of tissues where the functioning of feedback loops may be observed.** We used data from the GTEx project (GTEx Consortium, 2020) extracted from the Expression atlas (https://www.ebi.ac.uk/gxa/home). Examples of tissues or organs where the expression level of each gene involved in a particular feedback loop was at least 10 TPM were selected.

**Analysis of the evolutionary characteristics of genes.** The evolutionary characteristics of genes were evaluated using phylostratigraphic age index (PAI). PAI values were calculated for 19,556 human protein-coding genes using the Orthoscape software tool (Mustafin et al., 2017) as was described in (Mustafin et al., 2021).

## Results

The gene network regulating cholesterol biosynthesis

**Fig. 2.**
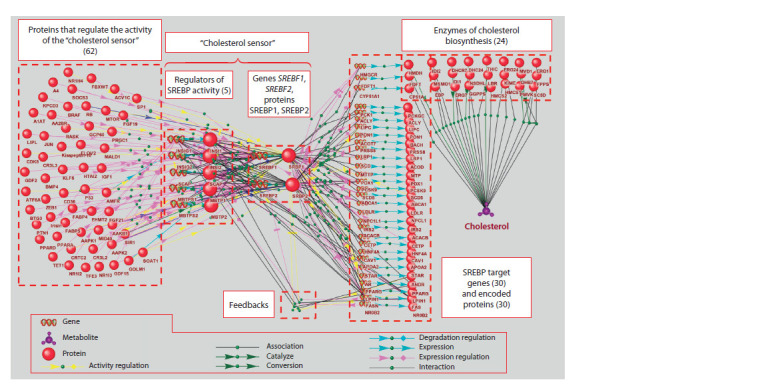
The gene network regulating cholesterol biosynthesis, visualized by ANDVisio. The ANDVisio program designates SREBP1 and SREBP2 as SRBP1 and SRBP2. Lists of genes from each functional group are presented in Supplementary Materials 1‑4. Supplementary Material 3 contains one more target gene (i. e. 31 genes), in the Figure this 31st gene (SREBF2) is placed in the group of objects designated as the “cholesterol sensor”.

At the first step, the so-called “small gene networks” were built using the ANDVisio program (as was described in “Materials and methods” and Supplementary Material 5). Next, the “small gene networks” were merged using the ANDVisio program. Thus, a gene network regulating cholesterol biosynthesis was constructed (Fig. 2). This network included: (1) the SREBF1 and SREBF2 genes and the proteins encoded by them; (2) five proteins regulating the activity of the SREBP1 and SREBP2 factors (INSIG1, INSIG2, SCAP, MBTPS1/S1P MBTPS2/S2P), and the genes encoding them (“cholesterol sensor”); (3) 62 proteins regulating the activity of genes and proteins of the “cholesterol sensor” (“regulatory proteins”); (4) 31 SREBP target genes (including SREBF2 itself) and the proteins encoded by them; (5) 243 interactions between objects (Fig. 2).

Feedback loops involving transcription factors from the SREBP subfamily

**Fig. 3.**
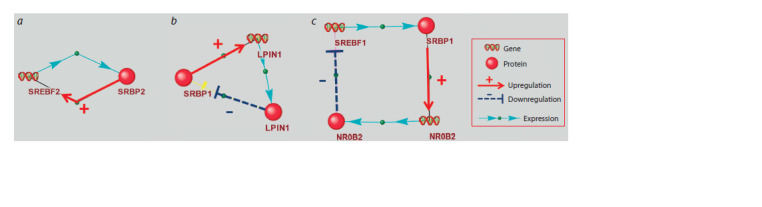
Feedback loops involving factors from the SREBP subfamily (indicated as SRBP1 and SRBP2). a – positive autoregulation of SREBF2 gene expression; b – a feedback loop involving the LPIN1 gene and the encoded protein; c – a feedback loop involving the NR0B2/SHP1 gene and the encoded protein.

**Feedback loops involving transcription factors from the SREBP subfamily with length 2, 3, and 4.** These feedback loops are shown in Figure 3. The factors from the SREBP subfamily are indicated in Figure 3 as SRBP1 and SRBP2. One of the three feedback loops shown in Figure 3 is positive and two feedbacks are negative.

SREBP2 (protein) → SREBF2 (gene) → SREBP2 (protein). The shortest feedback loop, which included two objects (Fig. 3a), was revealed when examining the list of SREBP target genes (Supplementary Material 3). According to R. Sato and co-authors, the promoter of the human SREBF2 contains SREBP2 binding site (Sato et al., 1996), mediating positive autoregulation of SREBF2 gene expression.

The search for feedback loops involving SREBPs was based on templates No. 1–4 presented in Supplementary Material 7. As a result, two feedbacks involving SREBP1 were found (Fig. 3b, c). No loops involving SREBP2 were found.

SREBP1 (protein) → LPIN1 (gene) → LPIN1 (protein) → SREBP1 (protein) (Fig. 3b). This is a negative feedback loop involving the LPIN1 gene (lipin 1) and the encoded protein. The promoter of the human LPIN1 contains the sterol regulatory element, and this element is responsible for the transcription activation of LPIN1, mediated by SREBP1 (in the Figure it is indicated as SRBP1) (Ishimoto et al., 2009). The LPIN1 protein suppresses the activity of SREBP1, preventing SREBP1 from binding to regulatory regions of its target genes, including the LPIN1 gene itself (Mateus et al., 2021). This mechanism is realized by regulating the SREBP1 transport inside the nucleus by the LPIN1 protein. LPIN1 promotes SREBP1 translocation to the nuclear lamina, where SREBP1 is inactivated (Peterson et al., 2011). The activity of LPIN1 is controlled by the mTOR kinase, which is involved in the response to growth factors (Peterson et al., 2011). Thus, the existence of a feedback loop involving LPIN1 indicates that the amplitude of transcriptional response to SREBP1 may be affected by growth factors.

SREBP1 (protein) → NR0B2/SHP1 (gene) → NR0B2/ SHP1 (protein) → SREBF1 (gene) → SREBP1 (protein) (Fig. 3c). This feedback loop involves the NR0B2/SHP1 gene and the encoded protein (SHP1, small heterodimer partner). The human NR0B2/SHP1 gene transcription is activated by SREBP1 (in the Figure it is indicated as SRBP1) (Kim et al., 2004). According to the UniProt Knowledge base (UniProt_ID = NR0B2_HUMAN), SHP1 is a transcription corepressor, it interacts with a number of transcription factors, preventing their activation by ligands. Thus, liganddependent transcription factors LRH-1, LXR and RXR may activate SREBF1 gene transcription, but the SHP1 protein prevents this activatory effect (Watanabe et al., 2004). Thus, the existence of a regulatory loop involving NR0B2/SHP1 and the encoded protein indicates that the transcriptional response to decreased cholesterol levels may be affected by other low molecular weight hydrophobic substances, which are ligands of transcription factors LRH-1, LXR, RXR and corepressor NR0B2/SHP1.

**Fig. 4.**
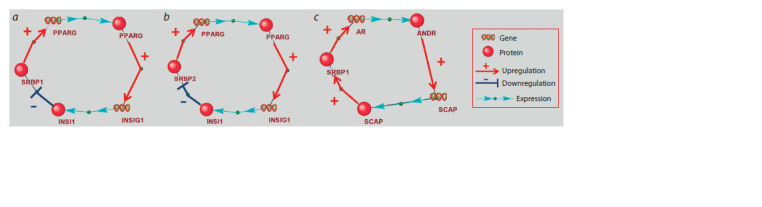
Feedback loops involving factors from the SREBP subfamily (designated as SRBP1 and SRBP2) and other genes and proteins functioning within the “cholesterol sensor”. a – feedback involving the SREBP1, PPARG and INSIG1 genes, as well as the encoded proteins; b – feedback involving the SREBP2, PPARG and INSIG1 genes, as well as the encoded proteins; c – feedback involving the SREBP1, AR and SCAP genes, as well as the encoded proteins.

**Feedback loops with length 5 involving factors from the SREBP subfamily, as well as proteins functioning within the “cholesterol sensor”.** We identified three regulatory circuits involving proteins functioning within the “cholesterol sensor”, which, in turn, affect the activity of SREBPs (Fig. 4). These feedback loops matched templates No. 7 and No. 8 presented in Supplementary Material 7. Two feedbacks included SREBP1 (indicated as SRBP1) (Fig. 4a, c) and one feedback loop included SREBP2 (indicated as SRBP2) (Fig. 4b). Two of the three regulatory loops are negative, and one is positive.

SREBP1 (protein) → PPARG (gene) → PPARG (protein) → INSIG1 (gene) → INSIG1 (protein) → SREBP1 (protein) (Fig. 4а).

SREBP2 (protein) → PPARG (gene) → PPARG (protein) → INSIG1 (gene) → INSIG1 (protein) → SREBP2 (protein) (Fig. 4b).

Two regulatory loops were found involving factors from the SREBP subfamily, as well as the PPARG and INSIG1 genes and encoded proteins. SREBP1 and SREBP2 (in Figures 4a and b these proteins are designated as SRBP1 and SRBP2) can interact with binding sites in the human PPARG promoter increasing transcriptional activity of PPARG (Fajas et al., 1999). PPARG is a transcription factor that can interact with the binding site (PPRE1) in the human INSIG1 promoter and activate transcription of the INSIG1 gene (Kast-Woelbern et al., 2004). This leads to increased expression of the INSIG1 protein, which retains preSREBP1 and preSREBP2 on the membrane of the endoplasmic reticulum, thereby suppressing translocation of preSREBPs to the Golgi apparatus, where SREBPs are activated by proteolytic processing (Roth et al., 2008).

SREBP1 (protein) → AR (gene) → ANDR (protein) → SCAP (gene) → SCAP (protein) → SREBP1 (protein) (Fig. 4c).

The promoter region of the human AR gene encoding the androgen receptor (in the Figure this protein is designated as ANDR) contains SREBP1 binding site. SREBP1 (in Figure 4c this protein is designated as SRBP1) binds to this regulatory element and activates the transcription of AR (Huang et al., 2010). The ANDR protein binds to the androgen response element in intron 8 of the human SCAP gene. This interaction leads to increased expression of SCAP (Heemers et al., 2004). In turn, SCAP escorts preSREBPs from endoplasmic reticulum to the Golgi apparatus where the SREBPs are activated (Guo et al., 2019). Thus, this is a positive feedback loop.

An examination of gene expression data from the GTEx project (GTEx Consortium, 2020) showed that the regulatory loops we found (Fig. 3 and 4) can function in a wide range of tissues. Examples of such tissues are given in Supplementary Materials 8 and 9.

The phylostratigraphic age of genes encoding enzymes of cholesterol biosynthesis and proteins functioning within the “cholesterol sensor”

The phylostratigraphic age index (PAI) was used to estimate the phylostratigraphic age of the genes. The PAI value indicates the evolutionary stage corresponding to the divergence stage of certain taxa. The PAI index takes values from 1 to 15 (Mustafin et al., 2021). The greater the PAI value of the studied gene, the younger the gene is.

**Fig. 5.**
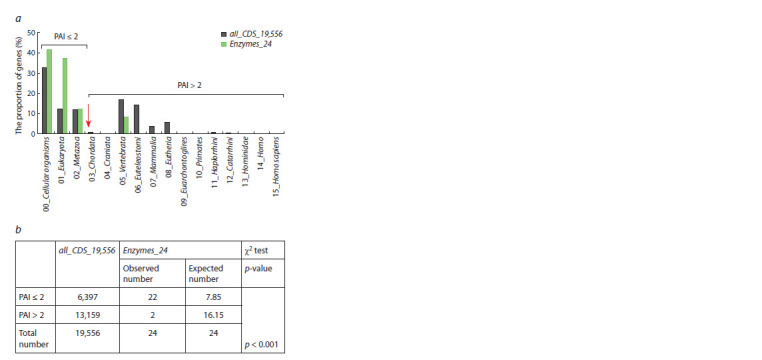
Phylostratigraphic age of human genes encoding enzymes of the cholesterol biosynthesis a – distribution of PAI values (indicated on the X axis) for all human proteincoding genes (control group of genes, designated as all_CDS_19,556, black columns) and genes encoding enzymes of the cholesterol biosynthesis (this group of genes is designated as Enzymes_24, green columns); b – according to the Chi-square criterion, the observed numbers of genes encoding enzymes and having PAI ≤ 2 differ from the expected numbers (p < 0.001).

**Genes encoding enzymes of cholesterol biosynthesis.** Figure 5 shows distributions by PAI values for all human protein-coding genes (black columns, control group) and 24 genes encoding enzymes of the cholesterol biosynthesis pathway (green columns). PAI values for genes encoding enzymes of the cholesterol biosynthesis pathway are presented in Supplementary Material 1. PAI values for the genes of the control group (designated as all_CDS_19,556) are unevenly distributed (Fig. 5a, black columns). Approximately one third of the genes (~33 %) had a PAI equal to zero (Cellular organisms, the root of the phytostratigraphic tree). And almost one fifth (17 %) of all protein-coding genes had a PAI value equal to 5 (the stage of Vertebrata divergence).

When considering the distribution of PAI values for a set of human genes encoding enzymes of cholesterol biosynthesis (Supplementary Material 1), it was found that 22 genes out of 24 (i. e. 92 %) had a PAI value <2 (Cellular organisms (the root of the phylostratigraphic tree) and the stages of Eukaryota and Metazoa divergence) (Fig. 5a, green columns). This number was different (p < 0.001) from the expected number (7.85) calculated based on the distribution obtained for a set of all human protein-coding genes containing 19,556 genes (Fig. 5b).

Thus, it turned out that the genes encoding enzymes of cholesterol biosynthesis are characterized by lower values of the PAI index compared to the set of all human proteincoding genes, that is, they are on average more “ancient”. This is in good agreement with the already known concepts. Firstly, cholesterol is found in ancient sedimentary rocks, and its derivatives are used as biological markers of past life on Earth (Simoneit, 2002). Secondly, it was found that the genes encoding enzymes of cholesterol biosynthesis were inherited by multicellular organisms from their last common eukaryotic ancestor (Zhang T. et al., 2019). In addition, it has been shown that enzymes involved in amino acid, carbohydrate and energy metabolism (including lipid metabolism) are highly conservative (Peregrín-Alvarez et al., 2009). This is due to the fact that the role of the enzyme is to interact with the substrate molecule, that is, the three-dimensional structures of the enzyme and the substrate must spatially fit each other. Therefore, as a rule, it is not the protein-coding, but the regulatory region of the gene encoding the enzyme that undergoes evolutionary changes.

**Genes encoding proteins functioning within the “cholesterol sensor”.** As mentioned above and shown in Figure 1, the “cholesterol sensor” is a set of proteins providing the regulation of the transcription of genes depending on the intracellular cholesterol level. The set of genes encoding proteins of this group includes: (1) the SREBF1 and SREBF2 genes encoding transcription factors; (2) the SCAP, INSIG1, and INSIG2 genes encoding proteins that change their conformational properties in response to changes in cholesterol levels, thereby regulating the rate of formation of active SREBPs; (3) the MBTPS1 and MBTPS2 genes encoding S1P and S2P proteases that cleave precursor proteins preSREBP1 and preSREBP2 (DeBose-Boyd, Ye, 2018; Jiang et al., 2020). The phylostratigraphic age of these genes indicates the ancient origin of their ancestral forms (see the color designations of objects in Figure 1, as well as Supplementary Material 2).

Four genes (SCAP, INSIG1, MBTPS1/S1P and MBTPS2/ S2P) have a PAI value equal to zero (Cellular organisms, the root of the phylostratigraphic tree). INSIG2 has a PAI value equal to 2 (the stage of Metazoa divergence). However, the SREBF1 и SREBF2 genes are younger. They have PAI values equal to 5 (the stage of Vertebrata divergence). Thus, although cholesterol was synthesized even in the most ancient organisms (Simoneit, 2002; Zhang T. et al., 2019), the molecu- lar mechanism controlling intracellular cholesterol level could have been formed at a later stage of evolution. This could have happened no earlier than the first vertebrates appeared.

**Fig. 6.**
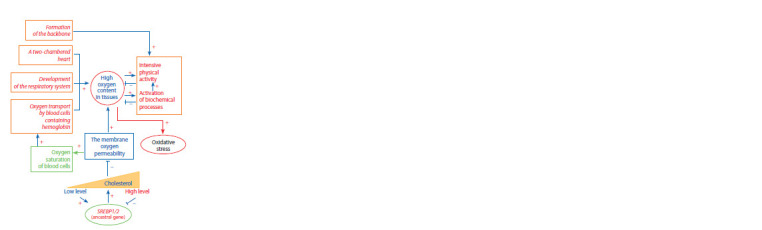
Characteristic features of the musculoskeletal, circulatory and respiratory systems, formed in animals at the evolutionary stage of Vertebrata divergence (shown in italics), and the significant role of cholesterol as a factor reducing oxygen permeability of the cell membrane.

The stage of Vertebrata divergence is characterized by a more complex organization of a number of physiological systems (Fig. 6). The formation of the backbone was accompanied by musculoskeletal system development and made it possible to move faster. As a result, the oxygen demand of muscles and other tissues increased. A twochamber heart was formed in vertebrates, which provided more efficient blood pumping and oxygen supply (Stephenson et al., 2017). At this stage of evolution, the respiratory system was being improved, and specialized oxygen-carrying blood cells (erythrocytes) arose (Snyder, Sheafor, 1999; Svoboda, Bartunek, 2015). The increased oxygen supply, on the one hand, contributed to the intensification of metabolic processes; on the other hand, it could cause oxidative stress.

The cell membrane cholesterol content affects the permeability of the membrane to oxygen: when cholesterol content is high, the membrane becomes more solid leading to reduced oxygen permeability (Zuniga-Hertz, Patel, 2019). This, on the one hand, protects cells from oxidative stress, but, on the other hand, inhibits the transport of oxygen to red blood cells and negatively affects the biochemical processes occurring with oxygen consumption. Thus, it became necessary to maintain the intracellular cholesterol level in an appropriate range. Since a certain evolutionary stage, this control was carried out by transcription factors from the SREBP subfamily

## Conclusion

This paper presents a gene network regulating cholesterol biosynthesis in human cells. The gene network systematizes data on: (1) the set of enzymes that carry out cholesterol biosynthesis; (2) proteins functioning within the “cholesterol sensor” (including transcription factors from the SREBP subfamily), this sensor is involved in the regulation of gene expression depending on the intracellular cholesterol level; (3) proteins regulating the activity of proteins functioning within the “cholesterol sensor”; (4) genes encoding proteins of these groups; (5) SREBP target genes. Feedback loops have been identified that control the activity of transcription factors from the SREBP subfamily, indicating the complex nature of the molecular genetic mechanisms that regulate cholesterol biosynthesis. In the future, we plan to expand the network by including higher-level regulatory effects (“regulators of regulators”). Such an extension will help to identify additional feedback loops controlling cholesterol biosynthesis

The analysis of the phylostratigraphic age of genes has shown that the ancestral forms of most human genes encoding enzymes of cholesterol biosynthesis and proteins of the “cholesterol sensor” could have been formed at early evolutionary stages (Cellular organisms (the root of the phylostratigraphic tree), as well as the stages of Eukaryota and Metazoa divergence). However, the phytostratigraphic age of genes encoding transcription factors of the SREBP subfamily corresponds to the stage of Vertebrata divergence. This fact indicates that the mechanism of gene transcription regulation in accordance with changes in cholesterol levels could have been formed at later evolutionary stages, that is, not earlier than the stage of Vertebrata divergence

## Conflict of interest

The authors declare no conflict of interest.
